# COVID-19 in healthcare workers in the state of Espírito Santo, Brazil: clinical and sociodemographic characteristics associated with death and hospitalization

**DOI:** 10.31744/einstein_journal/2022AO6241

**Published:** 2022-03-04

**Authors:** Rodolfo Antonio Corona, Arthur Arantes da Cunha

**Affiliations:** 1 Universidade Federal do Amapá Macapá AP Brazil Universidade Federal do Amapá, Macapá, AP, Brazil.

**Keywords:** Health personnel, Epidemiology, Occupational health, Coronavirus infections, COVID-19, SARS-CoV-2, Brazil

## Abstract

**Objective:**

To analyze clinical and sociodemographic characteristics associated with death and hospitalization of healthcare workers due to COVID-19, in addition to calculating the incidence rates per profession.

**Methods:**

A cross-sectional observational study using secondary open data from the State Health Department of Espírito Santo (ES), Brazil. The cases of COVID-19 in healthcare workers were recorded between February 27 and August 17, 2020, in Espírito Santo, excluding cases with missing information.

**Results:**

Of the confirmed cases, 75.6% (n=9,191) were female. The overall case fatality rate was 0.27% and the general hospitalization rate was 0.99%. The clinical outcome of death and the occurrence of hospitalization were associated with male sex, age ≥50 years, higher education, fever, difficulty breathing, cough, cardiac comorbidity, diabetes and obesity (p<0.05). Only the occurrence of hospitalization was associated with case reported in the metropolitan region of Vitória, runny nose, sore throat, headache and renal comorbidity (p<0.05). The profession with the highest incidence rate was nurse (16,053.2 cases/100,000 nurses).

**Conclusion:**

The study demonstrated high frequency of cases among women, low overall case fatality rate, and high incidence in nurses.

## INTRODUCTION

The pandemic of the new coronavirus affected the routines and habits of millions of people across the planet in 2020. First reported in Wuhan, China, in December 2019, severe acute respiratory syndrome coronavirus 2 (SARS-CoV-2) was identified as the pathogen causing coronavirus disease 2019 (COVID-19), defined by the World Health Organization (WHO).^(1,2)^ In the following months, the virus spread to all continents, reaching 188 countries by September 2020, and causing more than 1 million deaths worldwide.^(3)^ This high rate of dissemination was mainly attributed to the fact that the virus initially infects the cells of the respiratory system, allowing the affected individual to spread infectious viral particles through respiratory secretion (fomites, droplets, or aerosols) into the environment.^(4,5)^

In Brazil, COVID-19 was declared a public health emergency of national concern, by Ordinance # 188 of February 2020.^(6)^ The first case in the country was confirmed on February 26, 2020,^(7)^ and was followed by an accelerated process of community transmissibility, which in the subsequent months made Brazil the epicenter of the pandemic in Latin America, and the second country in the world with the highest number of deaths.^(8)^

Although it was possible to follow and analyze the evolution of the pandemic in other countries, Brazil had several problems facing viral dissemination. Among them, the lack of Personal Protective Equipment (PPE) for health workers in the frontline to fight infection stood out.^(9)^ Such equipment is essential for the worker’s safety and control of viral dissemination, both in Primary Care and in hospital settings.^(10)^

With the absence of vaccines and specific treatments proven to be efficient, the strategies of residential isolation and social distancing emerged as effective means to control the dissemination and, consequently, to maintain the flow of critical care, according to the care capacity of the Brazilian Public Health System (SUS - *Sistema Único de Saúde*).^(9)^ However, this measure, in many cases, is not suitable for health workers, because, in the context of the pandemic, there was an increased demand for these professionals.^(10)^

In this sense, healthcare professionals are a group at greater risk of being affected by COVID-19, since these workers have a higher degree of exposure to the virus, both in relation to contact with infected patients and viral load. Still, this workforce is not homogeneous, for there is a diversity of demographic characteristics and different levels and courses of professional training, which determine different forms and degrees of exposure.^(11)^ In addition to the constant concern of becoming ill, these professionals work under extensive stress loads, caused mainly by work overload, fear of infecting a family member, and lack of PPE and training to reduce the risk of infection during the care of patients suspected or confirmed to be infected with SARS-CoV-2.^(11,12)^

Therefore, the importance of understanding the patterns of involvement of COVID-19 in these professionals is evident. Knowledge of the characteristics that make up the profile of the affected worker, and the rates of involvement of different groups of workers, as well as clinical and demographic factors related to the worsening of the clinical picture, favor the implementation of specific infection control measures for certain professional groups, to mitigate the route of transmission of SARs-CoV-2 and protect the health of healthcare workers.

## OBJECTIVE

To analyze clinical and sociodemographic characteristics associated with death and hospitalization of healthcare workers due to COVID-19, and to calculate incidence rates by profession.

## METHODS

### Source of data and variables

This is a cross-sectional observational study, based exclusively on public, secondary, aggregated (without possibility of individual identification), and open access data, extracted from epidemiological bulletins^(13)^and the COVID-19 Panel - State of Espírito Santo (ES),^(14)^ produced by the State Health Department of Espírito Santo. The analysis was based on healthcare workers classified as confirmed cases through clinical diagnosis (cases of influenza-like illness (ILL) or severe acute respiratory syndrome (SARS) associated with anosmia or acute ageusia without previous cause, for which confirmation by another diagnostic method was not possible), clinical-epidemiological (cases of ILL or SARS, in which laboratory confirmation was not possible, but who had a history of close or household contact with an individual who had a laboratory diagnosis confirmed for COVID-19 in the 14 days prior to the onset of signs and symptoms), or laboratory tests (molecular biology test, with a “detectable” result for SARS-CoV-2, immunologic test with a “reactive” result for immunoglobulins M, A and/or G - IgM, IgA, and IgG, respectively, or antigen research test, with a “reactive” result for SARS-CoV-2).^(14)^ The variables related to the sociodemographic and clinical characteristics of the healthcare workers were extracted from the COVID-19 interactive panel of Espírito Santo.^(14)^ The numbers of infected health professionals and of registered healthcare workers in the state, by profession, were extracted from the epidemiological bulletins numbers 14 and 9, respectively.^(13)^

The period studied was defined from the date of notification of the first case of healthcare worker confirmed in the state (February 27, 2020) to the closing date of data collection for this research (August 17, 2020). Thus, the cases analyzed were registered between the 9^th^ and 34^th^ weeks of the 2020 epidemiological calendar of the Ministry of Health.

### Inclusion and exclusion criteria and procedures

**Figure 1:**
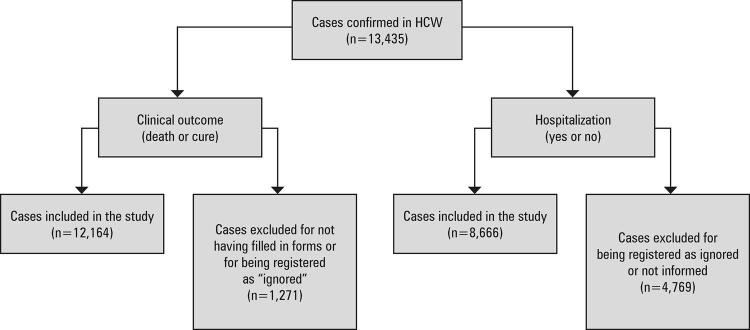
Flowchart of included and excluded cases of COVID-19 in healthcare workers

We included in the analysis the cases having information that showed certainty about the description of a particular variable. Cases that had data recorded as “ignored,” “not informed,” or that were not filled out were excluded (Figure 1). Especially for the clinical outcome (death or cure), this criterion excluded from the analysis possible active cases of the disease, avoiding an underestimation bias of case fatality. Thus, the lethality and hospitalization rates were calculated with different denominators, and each independent variable was evaluated with the number of cases, according to the availability of the data analyzed.

HCW: healthcare workers.

In addition, data on age range, location, and education variables were grouped, respectively, in less than 50 years, or 50 years or older; Metropolitan Region of Greater Vitoria or other locations; higher education or no higher education. The other variables were analyzed as they were in the database, considering the exclusion criteria.

The results, by variable and group (death and cure; hospitalization and non-hospitalization), were represented by absolute and relative numbers. The case fatality and hospitalization rates of healthcare workers affected by COVID-19 were calculated considering the number of deaths or hospitalizations of a certain variable and its respective total number of confirmed cases during the period. These rates were expressed as percentages.

The incidence rates (×100,000) of COVID-19 in the health professions analyzed were calculated based on the number of confirmed cases in a given occupation, and on the number of professionals registered in the state of Espírito Santo in the said profession.

### Data analysis

Statistical analysis was performed using the (SPSS) software, version 20.0. The continuous variable, age (years), was submitted to the Levene test to verify the homogeneity of variances, and to the Kolmogorov-Smirnov test to check data distribution. Based on these analyses, we chose to evaluate the possible difference between the groups (death *versus* cure; hospitalization *versus* no hospitalization) using the Mann-Whitney U test for two independent samples.

To compare the sociodemographic and clinical characteristics of healthcare workers affected by COVID-19 (categorical variables) with the clinical outcomes (cure or death) and the need for hospitalization or not, bivariate analysis was performed using the χ^2^ test for independence, or Fisher’s exact test. In this study, the significance level adopted was p≤0.05.

## RESULTS

### Difference between ages, according to clinical outcome and hospitalization

During the period analyzed, 13,435 confirmed cases of COVID-19 were reported in healthcare workers in the state of Espírito Santo. Of this total, 12,164 patients had registered information related to clinical outcome, in which 33 (0.27%) professionals died and 12,131 (99.73%) were cured. The mean age of individuals with the clinical outcome of death was 55.36±14.07 years, while the mean age of cure was 38.62±10.49 years (Table 1).

**Table 1:** Results of the statistical analyses to compare ages of healthcare workers with COVID-19, as per clinical outcome (death or cure) and hospitalization or not

Clinical outcomes									

All cases (n=12,164)			Death (n=33)			Cure (n=12,131)			p value^**†**^
		
Mean	SD	Median	Mean	SD	Median	Mean	SD	Median	
38.67	±10.54	38.00	55.36	±14.07	58.00	38.62	±10.49	38.00	<0.01^*^
**Hospitalizations**									

**All cases (n=8,666)**			**Hospitalized (n=86)**			**Non hospitalized (n=8,580)**			
			
**Mean**	**SD**	**Median**	**Mean**	**SD**	**Median**	**Mean**	**SD**	**Median**	
38.70	±10.59	38.00	50.10	±13.77	49.00	38.59	±10.49	38.00	<0.01^*^

Source: Governo do Estado do Espírito Santo. Superintendência Estadual de Comunicação Social do Espírito Santo (SECOM). Painel COVID-19 - Estado do Espírito Santo. Vitória: SECOM; 2021 [citado 2020 Out 4]. Disponível em: https://coronavirus.es.gov.br/painel-covid-19-es^(14)^

^†^ Mann-Whitney’s test; * a significant difference was observed.

SD: standard deviation.

Regarding hospitalizations, 8,666 health professionals had information regarding the need for hospitalization. Of these, 8,580 (99.01%) professionals did not need hospitalization, while 86 (0.99%) required this type of care. The mean age of those who were not hospitalized was 38.59±10.49 years, and the mean age of those hospitalized was 50.10±13.77 years.

### Sociodemographic and clinical characteristics associated with death

The group of female healthcare workers had the highest number of confirmed cases (75.6%). However, it was evident that the highest case fatality rate was found in the male group (0.57%), as well as the association of the clinical outcome death with the respective sex (p<0.01) (Table 2).

**Table 2:** Clinical outcome (death or cure) of healthcare workers with COVID-19 and respective case-fatality rates, according to sociodemographic and clinical characteristics

Variable	Outcome		Total	Case fatality rate %	p value^**†**^

	Death	Cure			
Total (n=12,164)	33 (100)	12,131 (100)	12,164 (100)	0.27	
Sex (n=12,163), n (%)					
Male	17 (51.5)	2,955 (24.4)	2,972 (24.4)	0.57	<0.01^*^
Female	16 (48.5)	9,175 (75.6)	9,191 (75.6)	0.17	
Age range, years (n=12,164), n (%)					
<50	12 (36.4)	10,177 (83.9)	10,189 (83.8)	0.12	<0.01^*^
50 or older	21 (63.6)	1,954 (16.1)	1,975 (16.2)	1.06	
Race/skin color (n=10,294), n (%)					
White	17 (54.8)	4,684 (45.6)	4,701 (45.7)	0.36	0.717
Brown	11 (35.5)	4,160 (40.5)	4,171 (40.5)	0.26	
Black	1 (3.2)	810 (7.9)	811 (7.9)	0.12	
Others	2 (6.5)	609 (5.9)	611 (5.9)	0.33	
Location (n=12,164), n (%)					
Metropolitan Region of Greater Vitória	15 (45.5)	7,423 (61.2)	7,438 (61.1)	0.20	0.064
Other locations	18 (54.5)	4,708 (38.8)	4,726 (38.9)	0.38	
Education (n=10,134), n (%)					
2No higher education	10 (38.5)	5,979 (59.2)	5,989 (59.1)	0.17	0.032^*^
Higher education	16 (61.5)	4,129 (40.8)	4,145 (40.9)	0.39	
Symptoms					
Fever (n=12,142)	27	5,621	5,648	0.48	<0.01^*^
Respiratory distress (12,142)	19	2,269	2,288	0.83	<0.01^*^
Cough (n=12,141)	25	7,009	7,034	0.36	0.038^*^
Runny nose (n=12,143)	11	5,639	5,650	0.19	0.128
2Sore throat (n=12,142)	7	4,246	4,253	0.16	0.096
Diarrhea (n=12,143)	4	2,035	2,039	0.20	0.472
Headache (n=12,140)	20	7,307	7,327	0.27	0.976
Comorbidity^‡^/risk factor					
Pulmonary comorbidity (n=12,127)	2	410	412	0.49	0.309
Cardiovascular comorbidity (n=12,130)	17	1,381	1,398	1.22	<0.01*
Renal comorbidity (n=12,128)	0	29	29	0.00	0.924
2Diabetes (n=12,127)	7	416	423	1.65	<0.01*
2Smoking (n=12,127)	0	149	149	0.00	0.665
2Obesity (n=12,090)	6	526	532	1.13	<0.01*

Source: Governo do Estado do Espírito Santo. Superintendência Estadual de Comunicação Social do Espírito Santo (SECOM). Painel COVID-19 - Estado do Espírito Santo. Vitória: SECOM; 2021 [citado 2020 Out 4]. Disponível em: https://coronavirus.es.gov.br/painel-covid-19-es^(14)^

^†^ independence χ^2^ test or Fisher’s exact test; * variables have statistically significant association; ^‡^ presence of coexisting diseases in relation to the disease that was the aim of the study.

Regarding age, it was found the highest number of healthcare workers affected were aged under 50 years (83.8%). Moreover, there was an association of clinical outcome death (63.6%) with the group aged 50 years or older (p<0.01) and a case fatality rate approximately nine times higher than the younger group.

As for education, most healthcare workers (59.1%) did not have higher education, and there was an association between the clinical outcome death and the fact of having higher education (p=0.032). Regarding symptoms, there was an association between presence of symptom and the clinical outcome death in the groups that had fever (p<0.01), respiratory distress (p<0.01), and cough (p=0.038). As for comorbidities and risk factors, we identified an association with the clinical outcome death and the existence of cardiac comorbidities (p<0.01), diabetes (p<0.01), and obesity (p<0.01).

### Sociodemographic and clinical characteristics associated with hospitalization

The absolute number of hospitalizations was higher among female healthcare workers (58.1%), but the highest hospitalization rate was among male workers (1.75%). Moreover, there was an association between male sex and hospitalization (p<0.01). Regarding the age group, although a higher number of hospitalizations was identified among healthcare workers under 50 years (51.2%), the hospitalization rate was higher (2.96%) in the group aged 50 years or older, and there was an association between this group and hospitalization (p<0.01) (Table 3).

**Table 3:** Hospitalization rates according to the sociodemographic and epidemiological characteristics of healthcare workers hospitalized due to COVID-19

Variable	Hospitalization		Total	Hospitalization rate %	p value^**†**^

	Yes	No			
Total (8,666)	86 (100)	8,580 (100)	8,666 (100)	0.99	
Sex (n=8,655), n (%)					<0.01*
Male	36 (41.9)	2,019 (23.6)	2,055 (23.7)	1.75	
2Female	50 (58.1)	6,550 (76.4)	6,600 (76.3)	0.76	
Age range, years (n=8,613), n (%)					<0.01*
<50	44 (51.2)	7,152 (83.9)	7,196 (83.5)	0.61	
50 or older	42 (48.8)	1,375 (16.1)	1,417 (16.5)	2.96	
Race/skin color (n=7,382), n (%)					0.573
White	30 (48.4)	3,271 (44.7)	3,301 (44.7)	0.91	
Brown	21 (33.9)	3,013 (41.2)	3,034 (41.1)	0.69	
Black	7 (11.3)	609 (8.3)	616 (8.3)	1.14	
Others	4 (6.5)	427 (5.8)	431 (5.8)	0.93	
Location (n=8,666), n (%)					0.028*
Metropolitan Region of Greater Vitória	64 (74.4)	5,398 (62.9)	5,462 (63.0)	1.17	
Other locations	22 (25.6)	3,182 (37.1)	3,204 (37.0)	0.69	
Education (n=7,459), n (%)					<0.01*
No higher education	29 (42.0)	4,378 (59.2)	4,407 (59.1)	0.66	
Higher education	40 (58.0)	3,012 (40.8)	3,052 (40.9)	1.31	
Symptoms					
Fever (n=8,666)	59	4,028	4,087	1.44	<0.01*
Respiratory distress (n=8,661)	43	1,579	1,622	2.65	<0.01*
Cough (n=8,661)	60	5,028	5,088	1.18	0.025*
Runny nose (n=8,662)	22	4,110	4,132	0.53	<0.01*
Sore throat (n=8,661)	20	3,026	3,046	0.66	0.024*
Diarrhea (n=8,661)	8	1,457	1,465	0.55	0.064
Headache (n=8,662)	40	5,253	5,293	0.76	<0.01*
Comorbidity^‡^/risk factor					
Pulmonary comorbidity (n=8,649)	4	297	301	1.33	0.351
Cardiovascular comorbidity (n=8,652)	32	1,056	1,088	2.94	<0.01*
Renal comorbidity (n=8,651)	2	17	19	10.53	0.015*
Diabetes (n=8,650)	19	308	327	5.81	<0.01*
Smoking (n=8,650)	1	121	122	0.82	0.657
Obesity (n=8,617)	13	401	414	3.14	<0.01*

Source: Governo do Estado do Espírito Santo. Superintendência Estadual de Comunicação Social do Espírito Santo (SECOM). Painel COVID-19 - Estado do Espírito Santo. Vitória: SECOM; 2021 [citado 2020 Out 4]. Disponível em: https://coronavirus.es.gov.br/painel-covid-19-es^(14)^

^†^ independence χ^2^ test or Fisher’s exact test; * variables have a statistically significant association; ^‡^ presence of coexisting diseases relative to the disease that was the aim of the study.

From the variables location and education, it was found that most hospitalizations occurred in the Metropolitan Region of Greater Victoria (74.4%) and among healthcare workers who had higher education (58.0%), there was an association between these variables (p=0.028 and p<0.01, respectively) and hospitalization in these groups. As for symptoms, we found an association between presence of symptoms and hospitalization in the groups that had fever (p<0.01), respiratory distress (p<0.01), cough (p=0.025), runny nose (p<0.01), sore throat (p=0.024), and headache (p<0.01). As to comorbidities and risk factors, the association of hospitalization was demonstrated with cardiac comorbidities (p<0.01), renal comorbidities (p=0.015), diabetes (p<0.01), and obesity (p<0.01).

### COVID-19 incidence rate by profession

Regarding the involvement of COVID-19 by profession, in absolute numbers, nurse technicians/licensed practical nurses had the highest number of cases (n=4,330). The highest incidence rate was found among nurses (16,053.2 cases/100,000 nurses) (Table 4).

**Table 4:** Incidence rate (×100,000) of COVID-19 in the most affected professions among healthcare workers

Profession	Number	Healthcare workers registered in the state of Espírito Santo	Incidence rate
Nurse technician/Licensed practical nurse	4,330	33,539	12,910.3
Nurse	1,496	9,319	16,053.2
Physician	1,148	11,382	10,086.1
Community Health Worker	501	4,917	10,189.1

Source: Governo do Estado do Espírito Santo. Superintendência Estadual de Comunicação Social do Espírito Santo (SECOM). COVID-19 - Boletins epidemiológicos. Vitória: SECOM; 2021 [citado 2020 Out 4]. Disponível em: https://coronavirus.es.gov.br/boletins-epidemiologicos^(13)^

## DISCUSSION

In this study of confirmed cases of COVID-19 in healthcare workers within the state of Espírito Santo, we found an overall case fatality rate of 0.27%. This rate is similar to that identified in a study of 1,716 Chinese healthcare workers (0.3%),^(15)^ and in another study of 8,845 North American healthcare professionals (0.3%).^(16)^ However, until October 1^st^, 2020, it is about ten-fold lower than the case fatality rate of the general population of Brazil (3.0%)^(17)^ and that of the general population of Espírito Santo (2.7%).^(14)^ In Mexico, Guerrero-Torres et al.^(18)^ described a case fatality rate of 5.1 between the general population (9.95%) and health professionals (1.95%), considering that most cases (88.9%) were hospitalized.^(18)^

In this sense, it should be considered that the increase in case fatality by COVID-19 is related to the age progression of the affected patients.^(4,15,16,18)^ Therefore, the low case fatality rate observed in the present study can be explained by the fact that most of these professionals are of working age. Moreover, by determination of the government of Espírito Santo,^(14)^ whenever possible, to remove or relocate health workers from the direct care of patients suspected or confirmed with COVID-19 in risk groups (60 years or older, with comorbidities, and pregnant women).^(14)^

Regarding hospitalization rates, it has been estimated that, in the general population, 20% of patients with COVID-19 would require hospitalization,^(19)^ but research has shown hospitalization rates of 12%^(20)^ and 14%.^(21)^ These rates, found in the general population, are higher than the hospitalization rate found in healthcare workers in the present study (0.99%). Possible justifications include age of the professionals, since the demand for this type of health care increases with age,^(4,15,16,18)^ and priority of testing given to these workers, which enables greater identification of less severe cases.^(16,18)^A higher hospitalization rate was observed in the Metropolitan Region of Greater Vitória (1.17%), possibly due to greater access to medical care in large centers.

As for age, an association was observed between advanced age and worsening of the clinical picture, in addition to higher case fatality and hospitalization rates in the group aged 50 years or older. Similar results were observed in the North American Burrer report,^(16)^which showed higher hospitalization and case fatality rates in professionals aged 55 years or older. In Brazil, epidemiological research exclusively on healthcare workers is still scarce, but the analyses related to characteristics of the general population diagnosed with COVID-19 also evidenced higher hospitalization and death rates among older individuals.^(4,21)^

Scientific literature relates several factors to the higher risk of worsening of the disease in people with older age and COVID-19. Most of these studies focus on the higher prevalence of comorbidities and on immune senescence.^(22)^ Among the biological alterations that might be related to the higher rates of hospitalization and case fatality in older professionals, the gradual decrease in the number of hair cells and in the lumen of the upper airways in aging stands out. Such changes might be partly responsible for the higher prevalence of respiratory symptoms in older people,^(23)^ which justifies the higher number of severe cases in this group.

AlGhatrif et al.^(24)^ demonstrated the role of angiotensin-converting enzyme 2 (ACE2) in the pathogenesis of COVID-19 and its relation to worsening cases, according to patient age, since this enzyme participates in the control of the immune response, especially in the respiratory system.^(24)^ It is also known that the amount of ACE2 is gradually reduced with aging.^(23)^ As to COVID-19, it has been shown that ACE2 is the entry receptor for SARS-CoV-2 in epithelial cells, and the enzyme is degraded by the virus after its internalization into the host cell, further reducing its activity in older people. This evidence leads to the hypothesis that older people would be more likely to manifest an exaggerated and uncontrolled tissue inflammatory response to SARS-CoV-2 infection, which could lead to worsening of symptoms, the need for hospitalization, and death.^(23,24)^

Furthermore, a great predominance of females was observed among the workers diagnosed with COVID-19 (75.6%), compatible with that identified by Moscola et al.^(25)^ (73.7%) and Guerrero-Torres et al.^(18)^ (61.1%). This corroborates the unequal proportion, worldwide, of the sexes in the health field, since women account for 70% of the workforce in the sector. In the region of the Americas, women account for 86% of nursing professionals.^(26)^

In this sense, it is noteworthy nursing professionals work in direct patient care and assistance, and are in greater contact with COVID-19 patients, which, associated with the scarcity of PPEs and lack of training for their correct use, observed especially at the beginning of the pandemic, may have increased the chance of infection.^(11,12,27)^ This fact, in part, may justify the results of the present study regarding the highest incidence rates of COVID-19, by profession, in nurse technicians, licensed practical nurses (12,910.3 cases/100 thousand), and registered nurses (16,053.2 cases/100 thousand). These incidence rates are respectively 4.9 and 6.0 times higher than those of the general population of Espírito Santo (2,639 cases/100 thousand),^(14)^ and 8.1 and 10.0 times higher than those of the general population of Brazil (1,600 cases/100 thousand),^(17)^ until the closing date of data collection in the present study.

As for the association between male sex and the clinical outcomes death and hospitalization, it should be noted other studies have found similar results in the general population,^(4,15,18)^ and in healthcare workers.^(18)^ The higher number of male deaths is apparently a pattern in nearly the entire planet, with ratios ranging from one female death for every 1.5-2.0 male deaths in the general population. The reason for this difference is probably multifactorial and encompasses factors based on immunology and genetics, lifestyle, hormonal factors, and prevalence of comorbidities.^(22)^

The analyses also demonstrated the existence of an association between worsening of the COVID-19 case and some clinical characteristics. These results are similar to those observed in other studies,^(16,21,28,29)^which also showed the association of respiratory and systemic symptoms and comorbidities, such as diabetes and cardiovascular diseases, with worsening of the clinical picture. It is noteworthy that, although the virus has tropism for the respiratory system, it can involve cells from other systems, such as the vascular, which also express ACE2.^(24,28)^

The invasion of multiple systems with tissue damage and functional impairment, added to deregulation of the immune response and the systemic release of cytokines, may in part explain the relation of respiratory symptoms, fever, and headache with worsening of the clinical picture. This immune panorama, observed in some patients with COVID-19, may also be related to the higher number of hospitalizations and deaths in patients with preexisting diseases,^(29,30)^ since comorbidities, such as diabetes and obesity, already affect the systemic immune response. Moreover, it is known that patients with comorbidities usually present with an older age.^(29,30)^Thus, not only complications related to preexisting diseases could aggravate the COVID-19 picture, but also age-related changes.

Finally, one of the limitations of this study is the fact that it is based on secondary data, which does not enable control of case reporting, thus not allowing the calculation of case fatality and hospitalization rates by profession, due to the unavailability of information, besides undercalculation of incidences due to underreporting.

## CONCLUSION

Respiratory and systemic symptoms, clinical comorbidities, sociodemographic characteristics, such as age, sex, education, and place of registration of the case, were associated with hospitalization and death of healthcare workers due to COVID-19. Females had a higher frequency of cases, but the case fatality and hospitalization rates in males were higher than in females. The overall case fatality rate in healthcare workers was about ten times lower than the rate in the general population of Brazil and Espírito Santo, during the same period. Still, nurses had a high incidence rate by profession.
